# Immune landscapes associated with different glioblastoma molecular subtypes

**DOI:** 10.1186/s40478-019-0803-6

**Published:** 2019-11-29

**Authors:** Maria Martinez-Lage, Timothy M. Lynch, Yingtao Bi, Carolina Cocito, Gregory P. Way, Sharmistha Pal, Josephine Haller, Rachel E. Yan, Amy Ziober, Aivi Nguyen, Manoj Kandpal, Donald M. O’Rourke, Jeffrey P. Greenfield, Casey S. Greene, Ramana V. Davuluri, Nadia Dahmane

**Affiliations:** 1 Department of Pathology, University of Pennsylvania School of Medicine, Philadelphia, PA 19104 USA; 2 Present address: Massachusetts General Hospital Department of Pathology, Harvard Medical School, Boston, MA 02114 USA; 3 William Penn Charter School, Philadelphia, PA 19144 USA; 4 Department of Neurosurgery, University of Pennsylvania School of Medicine, Philadelphia, PA 19104 USA; 5 Department of Preventive Medicine, Division of Health and Biomedical Informatics, Northwestern University Feinberg School of Medicine, Chicago, IL 60611 USA; 6 Present address: Abbvie Cambridge Research Center, Cambridge, MA 02139 USA; 7 Department of Neurological Surgery, Weill Cornell College of Medicine, New York, NY 10065 USA; 8 Department of Systems Pharmacology and Translational Therapeutics, University of Pennsylvania, Philadelphia, PA 19104 USA; 9 Present address: Imaging Platform, Broad Institute of MIT and Harvard, Cambridge, MA 02140 USA; 10 Department of Radiation Oncology, Dana-Farber Cancer Institute, Boston, USA; 11 Childhood Cancer Data Lab, Alex’s Lemonade Stand Foundation, Philadelphia, PA 19102 USA

## Abstract

Recent work has highlighted the tumor microenvironment as a central player in cancer. In particular, interactions between tumor and immune cells may help drive the development of brain tumors such as glioblastoma multiforme (GBM). Despite significant research into the molecular classification of glioblastoma, few studies have characterized in a comprehensive manner the immune infiltrate in situ and within different GBM subtypes.

In this study, we use an unbiased, automated immunohistochemistry-based approach to determine the immune phenotype of the four GBM subtypes (classical, mesenchymal, neural and proneural) in a cohort of 98 patients. Tissue Micro Arrays (TMA) were stained for CD20 (B lymphocytes), CD5, CD3, CD4, CD8 (T lymphocytes), CD68 (microglia), and CD163 (bone marrow derived macrophages) antibodies. Using automated image analysis, the percentage of each immune population was calculated with respect to the total tumor cells. Mesenchymal GBMs displayed the highest percentage of microglia, macrophage, and lymphocyte infiltration. CD68^+^ and CD163^+^ cells were the most abundant cell populations in all four GBM subtypes, and a higher percentage of CD163^+^ cells was associated with a worse prognosis. We also compared our results to the relative composition of immune cell type infiltration (using RNA-seq data) across TCGA GBM tumors and validated our results obtained with immunohistochemistry with an external cohort and a different method. The results of this study offer a comprehensive analysis of the distribution and the infiltration of the immune components across the four commonly described GBM subgroups, setting the basis for a more detailed patient classification and new insights that may be used to better apply or design immunotherapies for GBM.

## Introduction

There is a dynamic interaction between malignant and host cells within the tumor microenvironment, with host immune surveillance seeking to remove neoplasms and the tumor taking advantage of any opportunity to promote its own growth and progression. In many ways, the tumor microenvironment (TME) resembles the environment within chronic inflammation, as cancer cells interact with pericytes, fibroblasts, and immune cells [25]. These interactions shape the profile of cytokines, chemokines, growth factors, and soluble molecules that in turn shape the stroma, vasculature, and the tumor itself [46]. Although the presence of immune cells may seem indicative of a host response against the tumor, it has become clear that this is not necessarily the case.

To explain this, a three-step model of host-tumor interactions has been proposed, involving an “Elimination” phase, during which the host immune system tries to defeat the tumor, an “Equilibrium” phase whereby the host defense successfully controls tumor growth and curtails metastasis, and an “Escape” phase when tumor cells avoid surveillance to grow and spread freely [8]. Mechanisms for escape may include reprogramming the host immune system and interfering with the development, migration, and effector functions of immune cells to make them favorable to tumor progression [8]. Thus, understanding the immune cell composition of the tumor microenvironment is critical to understanding tumor progression and may provide insight on how to support the immune response and prevent evasion.

Glioma is the most common primary brain malignancy with approximately 20,000 new cases diagnosed every year in the United States [13]. According to the histological classification of the World Health Organization (WHO), gliomas can be divided into four different grades, with glioblastoma (GBM) being the most aggressive (Grade IV). GBM can arise de novo (primary GBMs), or derive from a lower-grade tumor (secondary GBMs) [27]. The standard of care for these tumors consists of surgical removal followed by radiation and chemotherapy. Despite aggressive treatment, GBM remains incurable, with a median overall survival of 12–15 months following diagnosis [1].

In the past few years, gene expression based studies have successfully identified transcriptional profiles that define the main GBM subclasses: proneural (PN), neural (N), classical (CL) and mesenchymal (MES) [32, 40]. Classical GBMs are also characterized by *Epidermal Growth Factor Receptor* (*EGFR)* amplification, lack of TP53 mutations, and oftentimes homozygous deletion of the *Cyclin Dependent Kinase Inhibitor* 2A (*CDKN2A)* [40]. Proneural GBMs, sometimes demonstrating alterations in *PDGFRA* pathway, are usually associated with a better outcome, particularly when harboring mutations in the one of the *Isocitrate Dehydrogenase* (*IDH)* genes [32, 40]. *IDH* mutations, in general, are also linked to higher CpG island methylation and usually have better clinical outcomes than GBMs with no *IDH* mutation [6, 42]. Mesenchymal GBMs are enriched in mesenchymal markers such as *YKL40* and *MET* [32] and co-mutation of the *Neurofibromin 1* (*NF1)* and *Phosphatase and TENsin homolog (PTEN)* genes, which parallels alterations seen in the epithelial-to-mesenchymal transition described in several other tumors [37, 40]. This subtype is commonly associated with a poor prognostic outcome [32]. Finally, neural GBMs strongly resemble the signature of neural cells, with strong expression of neuronal markers such as *NEEL*, *GABRA1*, *SYT1* and *SLC12A5* [40], to the point that subsequent studies suggest this group may have arisen from contamination from non-tumor resident cells in the initial analyses [41]. Overall, the mesenchymal and the proneural subtypes tend to be the most robust and reproducible signatures in GBM.

Given the differences between GBM subtypes, it is also important to understand whether there are corresponding differences in their immune landscapes. Initial analyses of the publicly available gene expression data from the Cancer Genome Atlas (TCGA) for GBM showed that mRNA expression for various cytokines, immune cell markers, and immune-associated signaling pathways were found to be increased in the mesenchymal subtype, suggesting that this subtype was the most proinflammatory [7]. Applying CIBERSORT [26] to TCGA GBM RNA-seq data and other publicly available datasets, Wang et al. found that both the M2 macrophage and neutrophil gene signatures were significantly associated with the mesenchymal subtype [41]. These findings, using gene expression, suggest that the GBM subtypes are different in their immune component; but they do not address immune phenotype heterogeneity, nor do they provide a quantitative analysis of the preponderance of immune cells in the tumor microenvironment.

Therefore, we performed a comprehensive immunohistologic screen to characterize the total percentage of cells within the tumor for the main immune populations (T/B lymphocytes and macrophage/microglia) across the four major GBM subtypes as previously described [32, 40]. We then performed statistical analyses to determine if the presence of certain immune infiltrates were correlated with overall survival. We found that mesenchymal GBM was the most immunogenic among the four subclasses while the proneural subtype was the least immunogenic. These results were complemented and validated by a computational analysis of RNA-seq data obtained on another cohort. Our analysis also revealed that the percentage of CD163^+^ macrophages in a glioma is the only statistically significant predictor of survival among the major immune infiltrates.

## Materials and methods

### Patient cohort

Ninety-eight cases of newly diagnosed GBMs (males *n* = 56, females *n* = 42) were reviewed by a board-certified neuropathologist (MML) and were used to generate 5 different TMA blocks. These tumors were all resected at the Hospital of the University of Pennsylvania under approved IRB and were stored as clinical fixed formalin paraffin embedded (FFPE) blocks in addition to fresh tissue banking per protocol. Patient classification and survival information in Additional file 1: Table S1.

### Generation of the tissue microarray (TMA)

For each case, all Hematoxylin and Eosin (H&E) stained slides from paraffin embedded blocks were examined, and representative areas of different histology were selected for inclusion in the TMA to account for the extreme morphological heterogeneity observed in some samples. Depending on the level of heterogeneity, as observed by histology, up to six cores for each tumor were included in the TMA blocks for a total of 315 unique tumor cores. The mean number of cores ± SD for each subtype were 3.29 ± 0.84, 3.12 ± 0.32, 3.18 ± 0.49, and 3.19 ± 0.62 for the classical, mesenchymal, neural, and proneural subtypes, respectively.

Brain control tissues (cerebral cortex, basal ganglia and cerebellar cortex) were included along the edges of the TMA, and four cores of tonsil tissue were also included as immune tissue control.

### Immunohistochemistry (IHC)

To detect various immune cells, we used the following antibodies: CD4 and CD8 for T lymphocytes, CD68 and CD163 for resident microglia and bone marrow derived macrophages, and CD20 for B lymphocytes (Additional file 1: Table S2). In order to support the reproducibility of our experiments we used antibodies routinely used in the clinical setting.

### Image acquisition and analysis

Images of each stained TMA slide were acquired at 10x magnification with the Vectra automated multispectral imaging system. Spectral libraries were generated from single stained slides using the Nuance FX multispectral imaging system software [23, 24, 36]. Spectral libraries and the scanned images were then loaded into inForm Advanced image analysis software (PerkinElmer) [21]. Automated image analysis was used to quantify the percentage of positive infiltrating cells for each immune cell marker (percentage positivity) based on a binary distribution of the 3,3' Diaminobenzidine (DAB) signal (positive/negative) which was determined for each slide by supervised analysis by a neuropathologist to address differences in staining intensity (Additional File 1: Table S3). Mutant IDH1 positivity was scored as positive/negative by manual interpretation of the scanned images.

### Quantification and statistical analysis of IHC

The results are displayed as median percentage ± mean absolute deviation (MAD). Statistical analysis was carried out with R (R Core Team 2013) and the threshold for statistical significance was set at *p* < 0.05. Differences among GBM subtypes were evaluated using one-way analysis of variance (ANOVA) for each of the cell type markers. Pair-wise differences among molecular subtypes were evaluated using student t-tests. Univariate overall survival analysis according to molecular subtypes status was evaluated by log-rank tests with Kaplan–Meier survival curves.

### Evaluation of concordance with TCGA data

We compared our results with expression-based estimates derived from TCGA. We estimated the proportions of selected cell types (CD4^+^, CD8^+^, and macrophages) using previously described marker genes [26]. We performed single sample gene set enrichment analysis (ssGSEA) [3] using an R implementation [15]. Data for TCGA were downloaded from UCSC Xena on August 16, 2016. These datasets contained clinical variables for 629 samples and gene expression measurements for 12,042 genes measured on 539 samples. We archived the data at the time of download on Zenodo [43]. Our analysis was performed via continuous analysis [4]. The computational environment is available from DockerHub (“gregway/gbm_immune_validation”). Source code is available on GitHub (https://github.com/greenelab/gbm_immune_validation), and the repository has been archived on Zenodo [44].

## Results

Most of the comprehensive analyses of the immune landscape in cancer, including those of glioblastoma, rely on RNA expression analyses of large datasets, i.e. TCGA [38]. While this has laid the foundation for understanding the immune component of cancer, these in silico approaches do not consider the distribution, localization or differences in proportions of immune cells within each tumor. To address this, we built tissue microarrays designed to comprehensively examine the composition of immune cells within the GBM microenvironment in situ.

### Digital image analysis of immune markers on GBM TMAs

We included primary tumors from 98 patients with GBM, obtained at the original surgery. These samples have previously been included in a cohort study of GBM subtypes using isoform-specific expression patterns [30]. GBM subclasses in this cohort were distributed as follows: 24.5% classical, 26.5% mesenchymal, 22.5% neural and 26.5% proneural (Table 1). *IDH1* R132H mutation as determined by immunohistochemistry (IHC) was extremely rare, only present in 4.1% of the cases. For each case, H&E stained slides were examined, and representative areas of different histology were selected to account for the heterogeneity observed in GBM samples with up to six cores for each tumor included in the TMAs.

**Table 1:** Characteristics of the GBM samples used in our cohort for TMAs generation

Variable	N	% of cohort	Median survival (months)	Log-rank Mantel-Cox test
**Gender**				*
Males	56	57.1	12	
Females	42	42.9	12	
**Age**				***
< 40	3	3.1	Undefined	
41–60	42	42.9	12	
61–80	48	48.9	11	
> 80	5	5.1	4	
**IDH1 R132H mutation**				***
Positive	4	4.1	Undefined	
Negative	94	95.9	12	
**Molecular subtype**				
Classical	24	24.5	12.5	
Mesenchymal	26	26.5	9.5	
Neural	22	22.5	15.5	
Proneural	26	26.5	10	

* *p* < 0.05; ** *p* < 0.01; *** *p* < 0.001; **** *p* < 0.0001

**Fig. 1:**
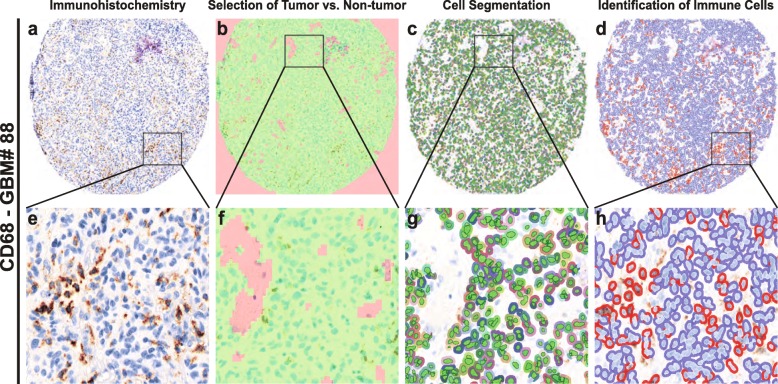
Automated cell analysis. **a** and **e**: Vectra scan multispectral image of a tissue core labeled with an immunohistochemical stain (CD68). **b** and **f**: Tissue segmentation. The image analysis software algorithm was trained to identify areas of tumor (green) and non-tumor (red), the latter of which includes areas of necrosis and vessels containing peripheral blood. **c** and **g**: Cell identification. The algorithm was trained to identify cells including tumor and infiltrating cells. Areas identified as non-tumor are not segmented. **d** and **h**: Automated binary scoring of immunohistochemical stain (blue – negative, red – positive)

In order to perform an unbiased analysis, we used a digital image analysis method (Fig. 1). First, we evaluated the spectral properties of the DAB stains, then we trained the software to identify tumor versus non-tumor areas. We excluded regions within vascular spaces and areas of necrosis. Next, we performed cell segmentation by training algorithms to identify unique cell types. We used the trained algorithms to identify and score immune infiltrating cells based on the DAB signaling spectral properties, thus avoiding investigator bias and providing increased accuracy and precision over traditional methods [23, 24, 36].

### Heterogeneity of the immune landscape in GBM

**Fig. 2:**
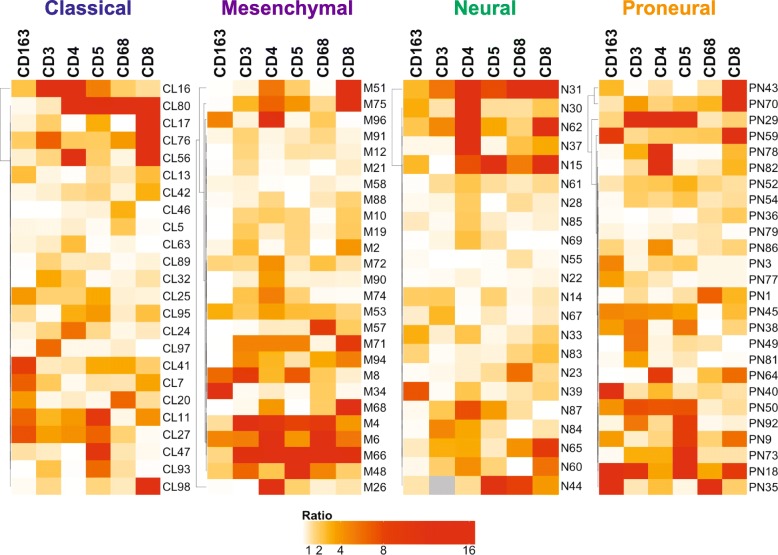
Immune heterogeneity is extensive across GBM subtypes. The heat maps show the heterogeneity in the immune marker expression across patient cores for each GBM subclass, with ratio breaks at 1, 2, 4, 8, and 16 (increasing from white to red). In all four subclasses, most of the cases displayed extensive heterogeneity in at least one of the immune markers studied among the cores. Grey square indicate that the data for this sample and specific marker was not examined for this analysis

**Fig. 3:**
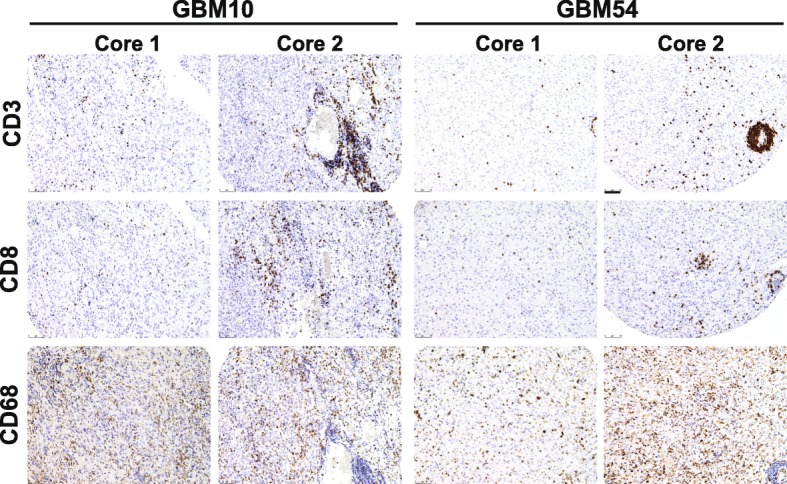
Heterogeneity in percentage of immune cells within the same GBM sample. In this figure**,** GBM10 and GBM54 are two representative examples of the heterogeneity in immune marker expression across different cores of the same GBM. GBM10, on the left, displays a strong heterogeneity in CD3 and CD8 infiltration between core 1 and core 2. Despite the overall lower levels in the immune infiltrate, GBM54, on the right, is also characterized by a substantial heterogeneity in CD3, CD8 and CD68. Scale bar 75 μm

A major gap in the biological understanding of GBM is a thorough characterization of the cellular and molecular heterogeneity of these tumors. This heterogeneity also affects treatment development. Thus, to obtain a more comprehensive view of the GBM immune landscape, we used several cores across all the sections obtained for each tumor. The choice for each core was made to represent at best the observed histological heterogeneity. In our cohort, the majority of the cases were characterized by heterogeneous infiltration of different immune cells. We then applied a semi-quantitative method to define the heterogeneity in the immune infiltrate across different cores in the same patient. For each patient, we calculated the ratios for each immune marker among cores, then averaged these values. The resulting ratios were plotted on a heatmap with ratio breaks at 1, 2, 4, 8, and 16, displayed with increasing color saturation from white to red (Fig. 2). The levels of intra-tumoral immune heterogeneity were not correlated with a specific immune population or with a specific GBM subtype. Interestingly, we observed that some samples were significantly more heterogeneous than others (Figs. 2 and 3). For example, GBM#31 showed significant differences in the percentage of most immune cell types (6/7) between the different cores (Fig. 2), while other tumors such as GBM#23 showed inter-core heterogeneity for only 1/7 immune cell types (Fig. 2). Visual inspection may also suggest that within subtypes there may be sub-clusters of samples with similar levels of heterogeneity. Though this analysis is subject to some bias in the selection of cores, it is clear that there are significant differences in heterogeneity of the immune landscape in GBM.

### GBM contains few B cells and the percentage of T cells varies across subtypes

To examine the contribution of lymphocytes to the GBM immune landscape, we compared percentages of CD20^+^, CD3^+^, CD5^+^, CD4^+^ and CD8^+^ cells relative to the global cellularity within the four molecular subtypes. We observed CD20^+^ B cells in only 4 cases out of all 98 GBMs (3 mesenchymal and one proneural (Additional file 1: Figure S1). Because of the low incidence of this cell population in our cohort, we decided not to include it in our analysis.

**Fig. 4:**
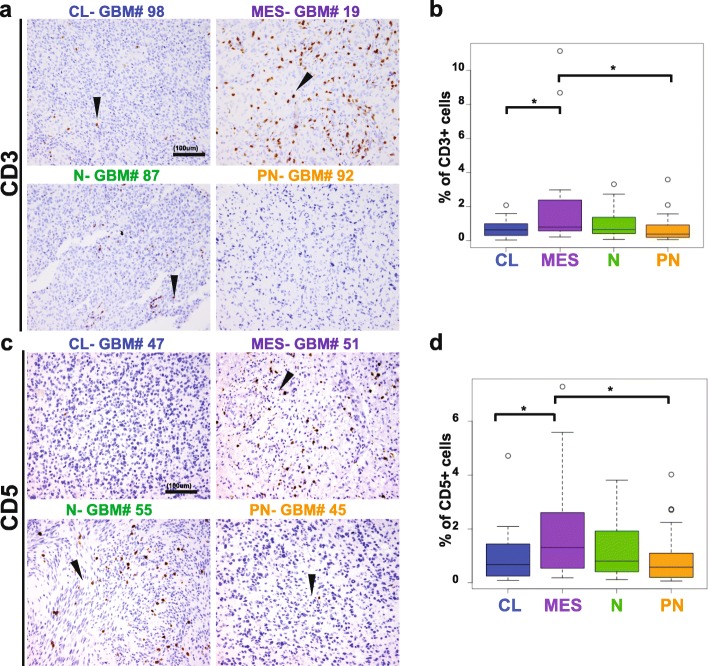
Levels of T cell infiltration differ across GBM subtypes. **a**, **c**: Representative classical, mesenchymal, neural and proneural GBM stained for CD3 and CD5 (in brown, arrows). **b**, **d**: Mesenchymal GBMs have the highest number of CD3 and CD5 positive T cells in comparison to the other molecular subtypes. Scale bar, 100 μm. Arrowheads indicate representative positive cells. CL, classical; MES, mesenchymal; N, neural; P, proneural. * *p* < 0.05; ** *p* < 0.01; *** *p* < 0.001; **** *p* < 0.0001

**Fig. 5:**
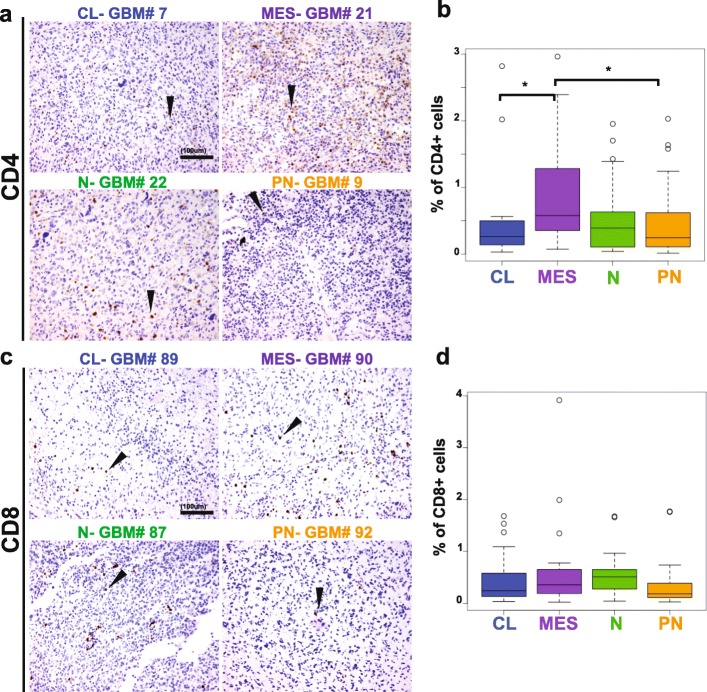
CD4^+^ but not CD8^+^ cells are differentially present in GBM subtypes. **a** and **c** show representative classical, mesenchymal, neural and proneural GBMs stained for CD4 and CD8 respectively. In **b**, mesenchymal GBM have a higher percentage of CD4 positive cells compared to the other GBM subtypes (of note, CD4 may be expressed in cells other than T lymphocytes, such as monocytes and macrophages). No differences were found in CD8 infiltrate (**d**). Scale bar, 100 μm. Arrowheads indicate representative positive cells. CL, classical; MES, mesenchymal; N, neural; PN, proneural. * *p* < 0.05; ** *p* < 0.01; *** *p* < 0.001; **** *p* < 0.0001

In order to determine the levels of infiltrating T lymphocytes, we compared both the percentage of CD3^+^ and CD5^+^ cells among all the GBM samples. Immunohistochemistry showed a perivascular and an infiltrative distribution of these cell subsets (Fig. 3), with both patterns present in most cases where vessels were present in the core. Despite variability among samples, on a whole, the mesenchymal subtype displayed the highest amount of T cell infiltration, both by CD3^+^ (0.77% ± 0.63) and CD5^+^ (1.31% ± 1.27). The percentage of CD3^+^ cells significantly differed between the mesenchymal and the proneural (0.32% ± 0.31, *p* = 0.036), as well as between the mesenchymal and the classical subtypes (0.57% ± 0.44, *p* = 0.03), but no other subgroups (Fig. 4). This was paralleled for CD5^+^ infiltrating T cells across the four different subtypes: significant differences were found between the mesenchymal and the proneural (0.34% ± 0.35, *p* = 0.023) and between the mesenchymal and the classical subtypes (0.67% ± 0.70, *p* = 0.0391). Furthermore, within the T cell population, the mesenchymal subtype also displayed a higher percentage of CD4^+^ cells (0.58 ± 0.59) in comparison to the classical (0.24% ± 0.23, *p* = 0.031) and the proneural subtypes (0.20% ± 0.18, *p* = 0.036) (Fig. 5). Surprisingly, no differences were found in the percentage of CD8^+^ infiltrating cells (ranging from 0.18% to 0.38% of the tumor area) among the four GBM subclasses (Fig. 5). Though it is important to note that lymphocytes constituted a very low percentage of the total cell number in the tumor overall (generally less than 5%), we found that mesenchymal GBMs tend to have the most T cell infiltration.

### Macrophages and microglia make the difference among the GBM subtypes

**Fig. 6:**
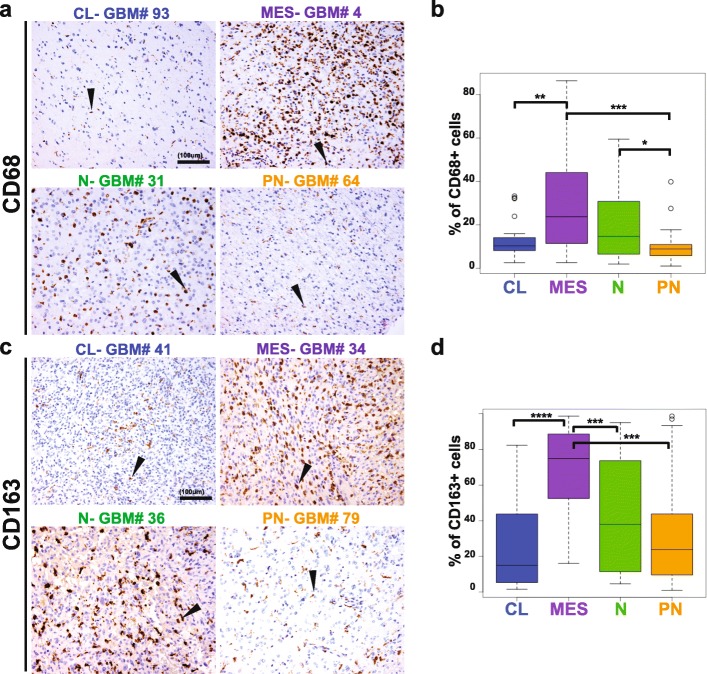
Macrophages and microglia are the predominant immune cells in GBM and their percentages differ across the molecular subtypes. **a** and **b** show classical, mesenchymal, neural and proneural GBMs stained for CD68 and CD163 (brown). In **c**, mesenchymal GBMs display the highest percentage of CD68 positive cells. In **d**, CD163 positive cells represent more than 80% of mesenchymal GBM cellularity, account this molecular subtype the most infiltrated by this cellular subtype. Significant difference in CD163 infiltrate was also observed between PN and CL GBMs. Scale bar 100 μm. Arrowheads indicate representative positive cells. CL, classical; MES, mesenchymal; N, neural; P, proneural. * *p* < 0.05; ** *p* < 0.01; *** *p* < 0.001; **** *p* < 0.0001

Among the immune cell types included in our study, macrophages and microglia were the predominant populations for every GBM subtype. In some cases, they constituted more than 80% of all cells present in the tumor (Fig. 6). As with the other cell types, quantitative analyses also showed great heterogeneity among and within examined samples. Across the GBM subtypes, CD163^+^ cells were the most abundant cell population in all subtypes, but there were significantly more frequent in mesenchymal samples (mesenchymal (78.94% ± 24.41) vs classical (43.02% ± 45.79), *p* < 0.001; vs proneural (25.44% ± 26.26), *p* = 0.003; vs neural (43.02% ± 45.79), *p* = 0.0007)) (Fig. 6). CD68^+^ cells were also most abundant in mesenchymal GBMs, and their percentage (23.75% ± 19.55) significantly differed compared with classical (10.21% ± 3.16, *p* = 0.0027) and proneural (7.60 ± 3.86% *p* = 0.0003) GBMs (Fig. 6).

The subtype with the next highest amount of macrophage and microglia infiltration was the neural subtype. Although no statistically significant results were observed for differences between the neural and proneural subtype for percentage of CD163^+^ macrophages, the percentage of CD68^+^ resident microglia significantly differed between neural (14.22 ± 13.60) and proneural (7.60 ± 3.86) subtypes (*p* = 0.012) (Fig. 6). Thus overall, we found a significantly higher percentage of tumor infiltrating macrophages and microglia in mesenchymal GBMs, followed by a higher percentage of tumor infiltrating microglia in the neural group over the classical and proneural subtypes.

### TCGA data shows significant differences in immune cell compositions across subtypes

**Fig. 7:**
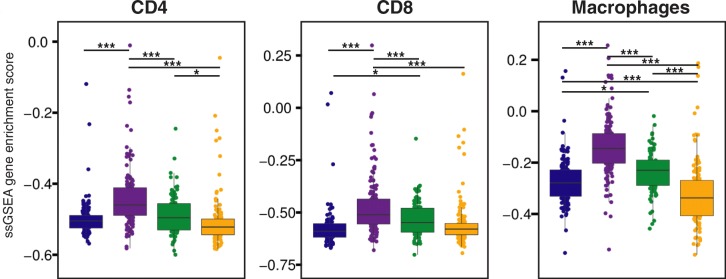
Relative amount of CD4^+^, CD8^+^, and macrophages cells across GBM subtypes. Single sample gene set enrichment scores for CD4, CD8, and macrophage gene sets defined by LM22 differentially expressed genes in the TCGA dataset. * *p* < 0.05; ** *p* < 0.01; *** *p* < 0.001; **** *p* < 0.0001

To complement and validate whether the results we observed in our single-institution cohort could be extrapolated to the GBM subtypes on a whole, we compared our results to the relative composition of immune cell type infiltration across TCGA GBM tumors (*n* = 539). We estimated the relative enrichment of CD4^+^, CD8^+^ and macrophage immune cell lineages in TCGA GBMs by applying ssGSEA stratified by gene expression-based subtype. We compared the ssGSEA empirical Cumulative Distribution Functions (eCDF) to the relative number of cells measured by percent positivity in our GBM dataset (Fig. 7). To determine the significance of the results, we fit three one-way analysis of variance (ANOVA) models comparing ssGSEA scores of CD4, CD8, and macrophage cell type markers, which were all highly significant (*p* = 2 × 10^− 16^, *p* = 6.28 × 10^− 15^ and *p* = 2 × 10^− 16^, respectively). To make subtype specific comparisons, we performed pairwise t-tests on ssGSEA enrichment scores comparing across TCGA GBM subtypes. The calculated *p* values were adjusted by false discovery rate (FDR) (Fig. 7). Macrophages constituted the main component of the immune cell infiltrate among all the subtypes and mesenchymal GBMs had the highest macrophage content (all *p* < 0.0001), followed by neural (all *p* < 0.05), classical (all *p* < 0.05), and proneural GBMs (all *p* < 0.0001). CD4^+^ and CD8^+^ T cell distribution followed the same trend observed in our IHC analysis, with the mesenchymal subtype having the highest infiltrate (all *p* < 0.0001).

Altogether, our IHC results were validated by an external cohort and a different method, showing that among the four GBM subtypes the mesenchymal subtype tends to have the highest immune cell content, while classical GBM seems to be less favorable for global immune cell infiltration. Further studies may be advised to investigate whether the molecular composition of each subclass has a role in promoting the invasion of the tumor by immune cells.

### Differences in the immune content and link to survival in GBM

**Fig. 8:**
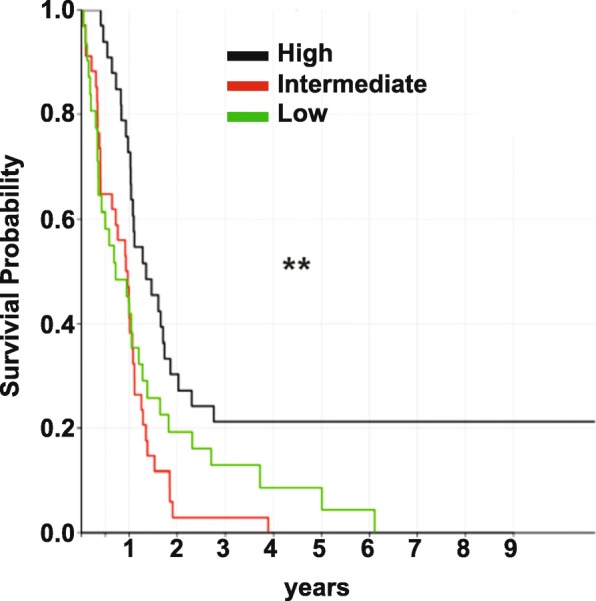
The percentage of CD163^+^ cells is associated to a poor prognosis. Intermediate (red) and high (green) levels of CD163 in GBM are correlated with a worse overall survival. **p < 0.01

To determine if the GBM immune content is correlated to survival, we plotted the result obtained for immune cell percentages on Kaplan-Meier curves (patients are equally divided into three groups based on percentages of each cell marker, i.e. low, medium, and high) (Fig. 8). Among the immune populations examined, the only one found to be significantly correlated to patient’s survival was the CD163^+^ macrophage lineage. Medium and high levels in percentage of CD163^+^ cells were indeed related to a worse prognosis (*p* = 0.00128). Interestingly, between the two conditions, medium levels of macrophage infiltration had a worse impact on patient survival than high levels. It is important to note however, that when we performed a cox regression model including age, sex and subtypes as covariates, CD163 had no significant effect, with *p*-value equal to 0.735.

## Discussion

This study is the first comprehensive immunohistochemical analysis of the immune microenvironment in the different subclasses of GBM. Despite an increasing interest in characterizing of the immune microenvironment in GBM, only gene expression data (RNA-seq) [5, 11, 41] and flow cytometry [11] have been used to characterize the subtypes to date. This has yielded invaluable insights into the immune profiles of these tumors, yet immunohistochemistry is still needed to validate these results and to understand the spatial relationship of these cells.

The interplay between tumor cells and their microenvironment is a dynamic process that strongly influences the progression and the outcome of several cancers. Given this, and the natural role of the immune system against neoplasia, the immune compartment of the tumor TME is an appealing target for potential therapies. Today, GBM remains the most common and lethal primary brain malignancy. Due to the pressing need to find alternative therapeutic approaches, as well as recent successes and failures in immunotherapy [16, 39] understanding its TME is critical. Furthermore, with the increased understanding of differences in biology and outcomes among subtypes, it is also necessary to understand the TME across subtypes.

Previous research has shown that the immune compartment of the TME is typically composed of several cell populations (lymphocytes, macrophages and dendritic cells) that may be associated with different outcomes. These immune parameters, including the density of cytotoxic and memory T cells at the center of the tumor and within tumor margins, were used to establish an “immunoscore” for colorectal cancer to predict recurrence and overall survival [12]. This was so successful that it has officially been proposed as a new element of the Tumor Node Metastasis (TNM) classification in colon cancer [29]. Correlations between various immune infiltrates and clinical outcomes have been shown for many cancers; however, the types of infiltrates and locations vary greatly among cancers [10]. Thus, it is crucial to determine whether an immunoscore may be developed with similar success in other cancers such as brain tumors.

Although the brain has been considered an “immune privileged” organ in the past [9], it has become clear that immune cell infiltrates constitute an important part of the TME in gliomas, with a significant fraction being macrophages. In brain tumors, tumor associated macrophages (TAMs) can be divided into two different populations: tissue resident microglia, which are derived from the yolk sac [14], and bone marrow-derived macrophages [34]. The latter derive from circulating monocytes and become particularly infiltrative during tumor progression when the integrity of the blood brain barrier is compromised [45]. Furthermore, M2 polarized TAMs have been associated with high grade gliomas and a more unfavorable outcome [18]. It has been proposed that they act via the expression of the transcription factor STAT3, whereby they produce cytokines to promote a tumor-supportive environment by suppressing the proliferation of anti-tumor CD4^+^ and CD8^+^ T cells and promoting the activity of regulatory CD4^+^ T cells [20]. Interfering with the pro-tumorigenic function of M2 macrophages has been shown to be effective in reducing glioma progression in mouse models and has been proposed as a therapeutic strategy [33, 35].

In our study, we found that GBMs demonstrate high levels of intra-tumor heterogeneity in immune infiltrate, that the GBMs subtypes vary significantly in the percentage of immune cells in their microenvironment, and that mesenchymal GBMs have the highest percentage of microglia, macrophage, and lymphocyte infiltration. Our results obtained using quantitative imaging analysis of IHC data are further in agreement with our analysis of immune cell components derived from the computational analysis of the TCGA GBM independent data set and extend on previous work describing gene expressions signature of various immune cells using RNA-seq [5, 11, 41].

Yet contrary to what has been described in other tumors, such as colon cancer [28], we did not observe any clear correlation between percentages of specific immune cells within the tumor and survival, besides for CD163^+^, which was related to a worse prognosis, although this may be due to its association with the mesenchymal subtype which carries worse prognosis. Though previous studies have found higher infiltrate of CD3^+^ cells (determined by cell percentage) to be associated with a better prognostic outcome [19] in glioblastoma and other solid tumors [10, 19, 31], this was not true in our cohort. We also did not find the CD8+ T cell infiltrate to be associated with longer survival, contrary to a histological study by Yang et al. [47]. This divergence may be related to the use of quantitative methods determine in the immune infiltrate, in our case we quantified the % of total cells while previous analysis quantified either number of cells/field or number of cells per area. Therefore, at this time we cannot generate an “immunoscore” to predict outcomes based on immune cell populations for the GBM subtypes [28].

A major benefit of our in situ study was the ability to observe the populations of cells and their spatial relationships. Our results revealed significant heterogeneity of GBM samples, both inter- and intratumorally. Though we found trends across the GBM subtypes for most cell types, there was also significant variation within the subtypes. Previous work has identified the mesenchymal GBM gene signature to be enriched in immunosuppressive genes, including a large proportion associated to the monocytic/macrophage lineage [7]. This suggests that a reciprocal interaction between TAMs and tumor cells may be crucial in the maintenance of the mesenchymal subtype. In line with this, our results demonstrate that the mesenchymal subtype has the highest degree of immune infiltration for all the populations evaluated. Thus, mesenchymal GBM may be the most immunogenic and may be suitable for immunotherapy. On the other hand, proneural GBMs, with their lower immune infiltration, may be closer to the immune “cold” category, which would require additional considerations for immunotherapy to be successfully applied.

One limitation of our study is that we only broadly characterized the main populations of immune cells. More specific types of cells, such as immunosuppressive T regulatory cells (Tregs), have been found to suppress the proliferation of other T cells and to favor tumor progression [2, 46]. In contrast, γδ T cells have been found to have HLA-independent cytotoxic activity [22]. Future analyses, using a combination of IHC and flow cytometry may be used to further classify our main cell populations into subclasses to better understand the immune microenvironment of these tumors.

In conclusion, our study is the first exhaustive immunohistochemical study of the immune infiltrate of the four GBM subtypes. We found a clear difference in immune infiltration between GBM subtypes, which was validated by gene-expression analysis of an external cohort. This offers new insight in intertumor immune cell heterogeneity. Though we only found one marker to be correlated with survival, it is possible that larger studies may be able to identify an “immunoscore” that could be used to predict disease progression and response to therapy.

## Supplementary information

**Additional file 1: Figure S1.** GBM tumors contain few B cells. **Table S1.** Clinical data and patient classification. **Table S2.** List of antibodies used in the study. **Table S3.** Results from Inform for each immune marker.

## Data Availability

N/A
