# Supplementary material for: Immune landscapes associated with different glioblastoma molecular subtypes
Source: Acta Neuropathol Commun. 2019 Nov 29;7:203. doi: 10.1186/s40478-019-0803-6 (PMC6902522; doi:10.1186/s40478-019-0803-6)
Supplement: Supplementary file 1 — Additional file 1: Figure S1. GBM tumors contain few B cells. Table S1. Clinical data and patient classification. Table S2. List of antibodies used in the study. Table S3. Results from Inform for each immune marker. [file 40478_2019_803_MOESM1_ESM.pdf]

Supplementary Materials

Supplementary Figure S1. GBM tumors contain few B cells.

The panel above shows representative images (B-E; B’-E’) from GBM stained for CD20 (in brown). Among all our cohort, CD20 positive B cells were only found in four cases. On the left, samples of Tonsil are used as controls (A, A’).

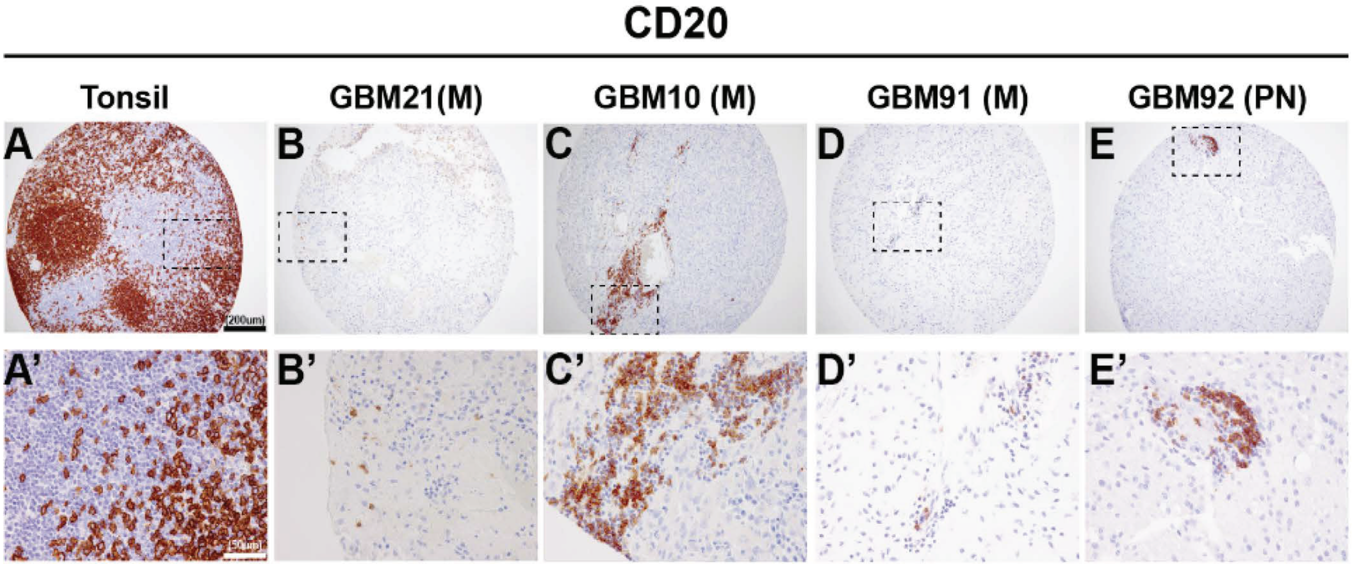

Supplementary Figure S1

Supplementary Table S1. Clinical data and patient classification.

TMA number	Subtype	Survival in months	IDH1 R132H
TMA1GBM1	PN	7	
TMA1GBM2	M	4	
TMA1GBM3	PN	>120	**POSITIVE**
TMA1GBM4	M	11	
TMA1GBM5	CL	11	
TMA1GBM6	M	4	
TMA1GBM7	CL	12	
TMA1GBM8	M	8	
TMA1GBM9	PN	4	
TMA1GBM10	M	12	
TMA1GBM11	CL	13	
TMA1GBM12	M	1	
TMA1GBM13	CL	20	
TMA1GBM14	N	>120	
TMA1GBM15	N	4	
TMA1GBM16	CL	3	
TMA1GBM17	CL	1	
TMA1GBM18	PN	7	
TMA1GBM19	M	28	
TMA1GBM20	CL	11	
TMA1GBM21	M	5	
TMA1GBM22	N	16	
TMA1GBM23	N	15	
TMA1GBM24	CL	22	
TMA1GBM25	CL	11	
TMA1GBM26	M	13	
TMA1GBM27	CL	28	
TMA1GBM28	N	>100	**POSITIVE**
TMA1GBM29	PN	4	
TMA1GBM30	N	24	
TMA1GBM31	N	74	
TMA1GBM32	CL	10	
TMA1GBM33	N	21	
TMA1GBM34	M	11	
TMA1GBM35	PN	6	
TMA1GBM36	PN	17	
TMA1GBM37	N	16	
TMA1GBM38	PN	>88	
TMA1GBM39	N	7	
TMA1GBM40	PN	>77	
TMA1GBM41	CL	16	
TMA1GBM42	CL	19	
TMA1GBM43	PN	12	
TMA1GBM44	N	15	
TMA1GBM45	PN	23	
TMA1GBM46	CL	12	
TMA1GBM47	CL	20	
TMA1GBM48	M	4	
TMA1GBM49	PN	>88	
TMA1GBM50	PN	0	
TMA1GBM51	M	6	
TMA1GBM52	PN	2	

Supplementary Table S1. Clinical data and patient classification.

TMA number	Subtype	Survival in months	IDH1 R132H
TMA1GBM53	M	4	
TMA1GBM54	PN	2	
TMA1GBM55	N	45	
TMA1GBM56	CL	13	
TMA2GBM57	M	19	
TMA2GBM58	M	12	
TMA2GBM59	PN	>70	
TMA2GBM60	N	3	
TMA2GBM61	N	8	
TMA2GBM62	N	12	
TMA2GBM63	CL	15	
TMA2GBM64	PN	4	
TMA2GBM65	N	>43	
TMA2GBM66	M	1	
TMA2GBM67	N	0	
TMA2GBM68	M	0	
TMA2GBM69	N	18	
TMA2GBM70	PN	13	
TMA2GBM71	M	1	
TMA2GBM72	M	12	
TMA2GBM73	PN	8	
TMA2GBM74	M	2	
TMA2GBM75	M	22	
TMA2GBM76	CL	22	
TMA2GBM77	PN	4	
TMA2GBM78	PN	12	
TMA2GBM79	PN	12	
TMA2GBM80	CL	13	
TMA2GBM81	PN	4	
TMA2GBM82	PN	4	
TMA2GBM83	N	10	
TMA2GBM84	N	13	
TMA2GBM85	N	33	
TMA2GBM86	PN	47	
TMA2GBM87	N	14	
TMA2GBM88	M	8	
TMA2GBM89	CL	4	
TMA2GBM90	M	60	
TMA2GBM91	M	32	
TMA2GBM92	PN	16	
TMA2GBM93	CL	5	
TMA2GBM94	M	11	
TMA2GBM95	CL	9	
TMA2GBM96	M	22	
TMA2GBM97	CL	15	
TMA2GBM98	CL	12	

Supplementary Table S2: List of antibodies used in the study.

Antibody	Company	Catalog #	Clone	Diluton
CD3	LEICA	PA0553	LN10	RTU
CD5	LEICA	PA0168		RTU
CD4	LEICA	PA0427	4B12	RTU
CD8	DAKO	M7103	C8/144B	1:40
CD68	LEICA	PA0273	514H12	RTU
CD163	LEICA	NCL-L-CD163		1:50
IDH	DIANOVA	DIA-H09	R132H	1:100
CD20	DAKO	IR60461	L26	RTU

Supplementary Table S3: Results from Inform for each immune marker.

TMA ID	CD68 Positivity	CD68 Tissue Category Area (Tumor %)	CD163 Positivity	CD163 Tissue Category Area (Tumor %)	CD3 Positivity	CD3 Tissue Category Area (Tumor %)	CD4 Positivity	CD5 Tissue Category Area (Tumor %)	CD8 Positivity	CD8 Tissue Category Area (Tumor %)
TMA1GMB1	0.271	0.6117	0.9645	0.4144	0.0034	0.6509	0.0022	0.6275	0.0027	0.6406
TMA1GMB1	0.0219	0.5748	0.9845	0.5104	0.0131	0.5831	0.003	0.523	0.0092	0.5544
TMA1GMB1	0.034	0.6136	0.8504	0.5394	0.013	0.635				
TMA1GMB2	0.0715	0.6313	0.1441	0.4159	0.0025	0.6393	0.0044	0.641	0.0016	0.6357
TMA1GMB2	0.0959	0.6273	0.182	0.6024	0.0162	0.6327	0.0035	0.6164	0.0122	0.6159
TMA1GMB2	0.078	0.6323	0.216	0.5097	0.002	0.6394	0.0035	0.6144	0.007	0.5789
TMA1GMB3	0.0057	0.5717	0.013	0.5966	0.0066	0.5868	0.0024	0.6082	0.0062	0.5838
TMA1GMB3	0.003	0.4301	0.0369	0.5214	0.0018	0.4412	0.0005	0.5957	0.0023	0.5798
TMA1GMB3	0.0201	0.6627	0.2399	0.6217	0.0326	0.6704	0.0013	0.6802	0.0075	0.6924
TMA1GMB4	0.4983	0.5893	0.9528	0.2444	0.0662	0.6478	0.0094	0.6301	0.0072	0.5448
TMA1GMB4	0.0398	0.701	0.302	0.6397	0.0018	0.7014	0.0006	0.6985	0.0006	0.7018
TMA1GMB4	0.0314	0.6881	0.4751	0.5678	0.0044	0.6893	0.0006	0.6797	0.0048	0.6754
TMA1GMB5	0.1713	0.3959	0.3451	0.1098	0.0154	0.3082	0.0035	0.5507	0.0013	0.2893
TMA1GMB5	0.4699	0.1952	0.4559	0.1934	0.0122	0.187	0.0023	0.5789	0.0015	0.5875
TMA1GMB6	0.0921	0.6891	0.1868	0.6394	0.0051	0.6986	0.0015	0.7161	0.0007	0.7076
TMA1GMB6	0.3193	0.4973	0.6269	0.6368	0.0439	0.7143	0.0101	0.7121	0.0196	0.6341
TMA1GMB6	0.0451	0.6932	0.0358	0.6595	0.0024	0.7013	0.0001	0.7039	0.0006	0.6488
TMA1GMB6	0.0391	0.7033	0.0417	0.7067	0.002	0.707	0.0003	0.6772	0.0012	0.6685
TMA1GMB6	0.004	0.5353	0.0912	0.2419	0.0054	0.4767	0.0081	0.4035	0.0025	0.2731
TMA1GMB7	0.1344	0.7026	0.1011	0.7153	0.0391	0.7258	0.0055	0.7178	0.0348	0.7283
TMA1GMB7	0.0546	0.5547	0.1015	0.5641	0.0076	0.605	0.0023	0.6656	0.004	0.6432
TMA1GMB7	0.067	0.729	0.0101	0.7399	0.0157	0.7446	0.0063	0.7255	0.0116	0.7268
TMA1GMB8	0.2034	0.4094	0.6109	0.5218	0.0135	0.6759	0.0011	0.6866	0	0.6711
TMA1GMB8	0.0881	0.6816	0.1431	0.6661	0.0031	0.6973	0.0037	0.682	0.0004	0.6891
TMA1GMB8	0.0505	0.6755	0.0503	0.5286	0.0007	0.6586	0.0005	0.6957	0.0002	0.6921
TMA1GMB9	0.0577	0.6701	0.1115	0.6704	0.0015	0.6803	0.0021	0.5694	0.0003	0.557
TMA1GMB9	0.1321	0.6781	0.4841	0.6393	0.0023	0.6787	0.0045	0.5301	0.0019	0.585
TMAGBM10	0.5326	0.4901	0.9685	0.3653	0.0548	0.6434	0.0067	0.6399	0.0172	0.636
TMAGBM10	0.4269	0.3848	0.9685	0.2299	0.0721	0.5874	0.0115	0.6394	0.0318	0.6295
TMAGBM10	0.4727	0.5342	0.9071	0.5447	0.2073	0.6532	0.0326	0.6533	0.1202	0.624
TMAGBM11	0.0217	0.7046	0.0044	0.7046	0.0017	0.6981	0.0002	0.6987	0.0006	0.6964
TMAGBM11	0.0163	0.6066	0.0183	0.614	0.0036	0.6213	0.0054	0.6244	0.0124	0.6094
TMAGBM11	0.0379	0.1613	0.1133	0.4801	0.013	0.3612	0.009	0.3662	0.0009	0.2744
TMAGBM12	0.0154	0.6212	0.2692	0.5545	0.0064	0.6554	0.0041	0.6397	0.0003	0.6048
TMAGBM12	0.0201	0.3753	0.2081	0.4175	0.0128	0.4646	0.0046	0.5009	0.0009	0.4783
TMAGBM12	0.0411	0.6538	0.2633	0.6127	0.0032	0.6709	0.0057	0.6687	0.0003	0.5956

Supplementary Table S3: Results from Inform for each immune marker.

TMA ID	CD68 Positivity	CD68 Tissue Category Area (Tumor %)	CD163 Positivity	CD163 Tissue Category Area (Tumor %)	CD3 Positivity	CD3 Tissue Category Area (Tumor %)	CD4 Positivity	CD5 Tissue Category Area (Tumor %)	CD8 Positivity	CD8 Tissue Category Area (Tumor %)
TMAGBM13	0.0547	0.6636	0.0109	0.6726	0.0009	0.6666	0.0001	0.6795	0.0002	0. 6877
TMAGBM13	0.1059	0.6235	0.0499	0.6387	0.0036	0.6372	0.0018	0.6356	0.0007	0.6342
TMAGBM13	0.1294	0.5001	0.0806	0.6114	0.0022	0.613	0.0007	0.6314	0.0001	0.6406
TMAGBM14	0.0682	0.6869	0.208	0.6008	0.02	0.6907	0.0035	0.6869	0.0112	0.6953
TMAGBM14	0.0311	0.5341	0.1474	0.5226	0.0085	0.5862	0.0046	0.5246	0.0042	0.5285
TMAGBM14	0.0666	0.6883	0.0577	0.6716	0.0051	0.6952	0.0043	0.6362	0.006	0.6701
TMAGBM15	0.0102	0.6214	0.0078	0.5441	0.008	0.3584	0.0001	0.5534	0.0017	0.3589
TMAGBM15	0.0006	0.6004	0.0087	0.614	0.003	0.6163	0.0028	0.4238	0	0.4651
TMAGBM15	0.1406	0.6032	0.9739	0.4563	0.0314	0.5833	0.0082	0.6267	0	0.6464
TMAGBM16	0.0906	0.6118	0.3708	0.6002	0.0008	0.6485	0.0004	0.6441	0.0011	0.6204
TMAGBM16	0.1364	0.3569	0.4532	0.1222	0.0047	0.2249	0.003	0.5283	0.0012	0.2135
TMAGBM16	0.0934	0.5101	0.2533	0.1371	0.056	0.1939	0.0037	0.4841	0.0034	0.2455
TMAGBM16	0.0381	0.7323	0.1584	0.7069	0.0001	0.7273	0	0.7077	0.0003	0.7075
TMAGBM16	0.025	0.7236	0.0465	0.6968	0.002	0.7341	0	0.7094	0.0017	0.704
TMAGBM16	0.0333	0.6738	0.2155	0.6152	0.0025	0.6752	0.0011	0.5937	0.0019	0.5852
TMAGBM17	0.1638	0.4824	0.3018	0.3727	0.0198	0.5523	0.0023	0.6376	0.0024	0.6329
TMAGBM17	0.1445	0.5745	0.79	0.5255	0.0047	0.5957	0.0009	0.5871	0	0.6109
TMAGBM17	0.168	0.5287	0.615	0.4996	0.0077	0.5692	0.0121	0.5753	0	0.5655
TMAGBM18	0.1915	0.1912	0.4751	0.2474	0.0413	0.2616	0.0185	0.596	0.0459	0.5951
TMAGBM18	0.0512	0.6325	0.0118	0.6399	0.0019	0.6358	0.0018	0.6543	0.0039	0.6575
TMAGBM18	0.0296	0.4818	0.1357	0.5113	0.004	0.5044	0.0116	0.6008	0.003	0.5903
TMAGBM19	0.3716	0.5878	0.7323	0.5281	0.0903	0.6259	0.0093	0.6034	0.0427	0.6124
TMAGBM19	0.3452	0.5289	0.9596	0.4525	0.1786	0.6187	0.0133	0.6207	0.1172	0.6277
TMAGBM19	0.5644	0.5048	0.7873	0.57	0.0466	0.6005	0.021	0.5799	0.0161	0.5971
TMAGBM19	0.5344	0.5851	0.7813	0.5782	0.0319	0.6177	0.0057	0.6118	0.0312	0.6322
TMAGBM20	0.1031	0.5988	0.0054	0.5876	0.0018	0.6032	0.0015	0.5888	0.0019	0.6082
TMAGBM20	0.0056	0.6213	0.0112	0.6306	0.0039	0.6289	0.0015	0.6208	0.002	0.6427
TMAGBM20	0.0611	0.5943	0.0853	0.9819	0.0017	0.693	0.0008	0.6754	0.0008	0.6935
TMAGBM21	0.8151	0.3243	0.782	0.6983	0.0156	0.7161	0.0251	0.6986	0.0021	0.7113
TMAGBM21	0.8505	0.0615	0.8261	0.4132	0.0246	0.6459	0.0164	0.628	0.0024	0.5871
TMAGBM21	0.8792	0.1382	0.8671	0.6077	0.0464	0.6655	0.0215	0.6335	0.0044	0.6137
TMAGBM22	0.667	0.398	0.4183	0.9783	0.0257	0.6848	0.014	0.6637	0.0037	0.6807
TMAGBM22	0.6088	0.2737	0.2441	0.9728	0.0398	0.6651	0.0208	0.6582	0.0066	0.6583
TMAGBM22	0.5082	0.375	0.6281	0.9232	0.0339	0.6814	0.0238	0.6692	0.0062	0.6787
TMAGBM23	0.8103	0.655	0.1393	0.9929	0.0094	0.748	0.0024	0.7595	0.0068	0.7364
TMAGBM23	0.0526	0.1317	0.0936	0.9937	0.0048	0.7372	0.0075	0.7419	0.0178	0.7297
TMAGBM23	0.2579	0.5625	0.2026	0.8991	0.0082	0.713	0.0024	0.6829	0.0043	0.6957

Supplementary Table S3: Results from Inform for each immune marker.

TMA ID	CD68 Positivity	CD68 Tissue Category Area (Tumor %)	CD163 Positivity	CD163 Tissue Category Area (Tumor %)	CD3 Positivity	CD3 Tissue Category Area (Tumor %)	CD4 Positivity	CD5 Tissue Category Area (Tumor %)	CD8 Positivity	CD8 Tissue Category Area (Tumor %)
TMAGBM24	0.0862	0.5821	0.0672	0.9458	0.004	0.6602	0.0117	0.627	0.001	0.6364
TMAGBM24	0.1547	0.6553	0.1508	0.9925	0.0051	0.6855	0.0028	0.652	0.0031	0.6705
TMAGBM24	0.1359	0.6444	0.129	0.9895	0.0016	0.6697	0.001	0.629	0.0027	0.6507
TMAGBM25	0.0781	0.5654	0.0094	0.9918	0.0014	0.6966	0	0.6727	0.0051	0.6716
TMAGBM25	0.0845	0.6172	0.0334	0.9929	0.0003	0.6821	0.0005	0.6656	0.0031	0.6759
TMAGBM25	0.1467	0.6439	0.0032	0.9971	0.0007	0.6865	0.0004	0.6469	0.0032	0.6818
TMAGBM26	0.3801	0.2089	0.5373	0.9007	0.0034	0.6706	0.0014	0.6392	0.0015	0.4543
TMAGBM26	0.3378	0.3529	0.645	0.8958	0.0063	0.6458	0.0098	0.6244	0.0013	0.4151
TMAGBM26	0.7826	0.0889	0.6155	0.7876	0.0039	0.6381	0	0.5311	0.0053	0.5268
TMAGBM27	0.1443	0.7142	0.344	0.9887	0.0117	0.7466	0.0048	0.7457	0.0102	0.7359
TMAGBM27	0.0278	0.7062	0.0226	0.9985	0.002	0.7399	0.001	0.7268	0.0099	0.7309
TMAGBM27	0.1015	0.6765	0.0466	0.9886	0.0021	0.7222	0.0006	0.7086	0.0126	0.7132
TMAGBM28	0.2314	0.5643	0.1396	0.9848	0.0023	0.6659	0.0005	0.6561	0.0035	0.6039
TMAGBM28	0.3324	0.4351	0.2417	0.9363	0.0059	0.6138	0.0043	0.5784	0.0013	0.5604
TMAGBM28	0.6799	0.2128	0.125	0.874	0.0044	0.4468	0.0045	0.5102	0.0026	0.4456
TMAGBM29	0.1797	0.6561	0.6763	0.9186	0.0015	0.6857	0.0013	0.6771	0.0009	0.6991
TMAGBM29	0.1025	0.6102	0.3771	0.9295	0.0101	0.6796	0.0016	0.6728	0.0021	0.6951
TMAGBM29	0.0829	0.6471	0.2231	0.9805	0.0003	0.6757	0	0.6429		
TMAGBM30	0.0267	0.5783	0.1219	0.9447	0.0016	0.6649	0.0017	0.6479	0.0212	0.676
TMAGBM30	0.2348	0.4946	0.0112	0.992	0.0029	0.3394				
TMAGBM30	0.0253	0.4799	0.0225	0.9745	0.0111	0.639	0	0.3654	0.0226	0.6268
TMAGBM30	0.282	0.6131	0.0278	0.993	0.0066	0.6604	0.0029	0.6523	0.0059	0.6583
TMAGBM31	0.396	0.3177	0.959	0.4228	0.028	0.667	0.025	0.647	0.0131	0.4874
TMAGBM31	0.1423	0.5956	0.9639	0.6973	0.0277	0.6782	0.0325	0.6686	0.0101	0.6074
TMAGBM31	0.3009	0.566	0.8654	0.6165	0.0125	0.6881	0.0099	0.6706	0.0041	0.6209
TMAGBM31	0	0.3226	0.1678	0.9387	0.0022	0.718	0.0007	0.6913	0.0002	0.7164
TMAGBM32	0.0846	0.6434	0.0545	0.9969	0.0035	0.6771	0.0005	0.6507	0.0024	0.6791
TMAGBM32	0.0903	0.651	0.0671	0.9955	0.0079	0.6855	0.0017	0.6572	0.0033	0.6828
TMAGBM32	0.0554	0.1755	0.0564	0.8497	0.0364	0.3703	0.0025	0.4855	0.0063	0.3702
TMAGBM33	0.1046	0.5791	0.0055	0.9854	0.0039	0.6347	0.0017	0.5894	0.0019	0.459
TMAGBM33	0.1186	0.5973	0.0073	0.9813	0.0047	0.6566	0.0067	0.6041	0.0068	0.6474
TMAGBM33	0.2295	0.5526	0.1847	0.956	0.0074	0.626	0.001	0.5922	0.0094	0.5775
TMAGBM34	0.2397	0.5586	0.3144	0.9359	0.0023	0.6675	0.0021	0.6699	0.0014	0.648
TMAGBM34	0.0735	0.359	0.0207	0.9743	0.0051	0.6178	0.0015	0.5973	0.0023	0.5795
TMAGBM34	0.2385	0.4923	0.3514	0.9214	0.0059	0.6982	0.0041	0.6994	0.0013	0.666
TMAGBM34	0.0502	0.6376	0.009	0.9946	0.0039	0.6878	0.0051	0.6777	0.0034	0.6938
TMAGBM35	0.0292	0.6968	0.0452	0.9932	0.0007	0.7769	0.0005	0.7801	0.0001	0.7797

Supplementary Table S3: Results from Inform for each immune marker.

TMA ID	CD68 Positivity	CD68 Tissue Category Area (Tumor %)	CD163 Positivity	CD163 Tissue Category Area (Tumor %)	CD3 Positivity	CD3 Tissue Category Area (Tumor %)	CD4 Positivity	CD5 Tissue Category Area (Tumor %)	CD8 Positivity	CD8 Tissue Category Area (Tumor %)
TMAGBM35	0.1407	0.322	0.2412	0.9408	0.0094	0.5022	0.004	0.4212	0.0028	0.4906
TMAGBM35	0.0034	0.5426	0.0023	0.991	0.002	0.6959	0.0005	0.6315	0.0004	0.6552
TMAGBM36	0.4107	0.4096	0.0216	0.9116	0.0039	0.6861	0.0023	0.6712	0.0021	0.6951
TMAGBM36	0.1744	0.5722	0.1995	0.956	0.0059	0.6653	0.002	0.6525	0.0011	0.6715
TMAGBM36	0.502	0.3526	0.1986	0.9462	0.0054	0.6382	0.0024	0.4741	0.0007	0.6201
TMAGBM36	0.1373	0.5875	0.0672	0.9897	0.0027	0.6808	0.0009	0.6677	0.0004	0.6933
TMAGBM36	0.1502	0.5508	0.3413	0.7202	0.011	0.6501	0.0038	0.6464	0.0004	0.6573
TMAGBM37	0.0587	0.6712	0.0572	0.9844	0.0053	0.7206	0	0.7106	0.0074	0.722
TMAGBM37	0.0737	0.663	0.0674	0.9938	0.0026	0.7266	0.0008	0.7273	0.0028	0.7342
TMAGBM37	0.0966	0.6335	0.0994	0.9936	0.0031	0.6878			0.002	0.3907
TMAGBM37	0.081	0.6706	0.1636	0.9751	0.0047	0.7038	0.0017	0.6825	0.0016	0.713
TMAGBM37	0.0127	0.6312	0.0868	0.9712	0.0028	0.6737	0	0.6525	0.0006	0.6792
TMAGBM38	0.0102	0.2767	0.0063	0.9334	0.0017	0.458	0.0007	0.4509	0.0004	0.4473
TMAGBM38	0.0447	0.6569	0.0004	0.9944	0.0013	0.6917	0.0004	0.6868	0.0013	0.6583
TMAGBM38	0.164	0.3793	0.0292	0.739	0.0278	0.5978	0.0089	0.5943	0.0135	0.4349
TMAGBM38	0.0397	0.3238	0.0036	0.9901	0.0009	0.6564	0.0034	0.6536	0.0067	0.6587
TMAGBM39	0.0435	0.4901	0.1698	0.9444	0.0257	0.6215	0.0007	0.6348	0.0007	0.6363
TMAGBM39	0.4021	0.1974	0.0091	0.9682	0.0122	0.6278	0.0135	0.6751	0.005	0.6508
TMAGBM39	0.2806	0.4149	0.0442	0.971	0.0294	0.6503	0.0017	0.6658	0.0048	0.6528
TMAGBM40	0.2058	0.3537	0.2164	0.924	0.0044	0.5902	0.0019	0.611	0.0031	0.619
TMAGBM40	0.0433	0.2225	0.0057	0.9743	0.0068	0.5957	0.0027	0.6144	0.0024	0.6115
TMAGBM40	0.1559	0.4652	0.1098	0.8595	0.0112	0.6262	0.0211	0.6388	0.0166	0.6224
TMAGBM41	0.7062	0.3463	0.5876	0.7373	0.0096	0.2634				
TMAGBM41	0.2175	0.2062	0.875	0.4579	0.0086	0.5854	0.0037	0.5799	0.0031	0.5486
TMAGBM41	0.1155	0.6627	0.058	0.9968	0.0036	0.6661	0	0.6845	0.0007	0.6798
TMAGBM41	0.0725	0.589	0.0273	0.9953	0.0024	0.6399	0.001	0.6509	0.0004	0.6497
TMAGBM41	0.0824	0.5088	0.2483	0.9163	0.0214	0.6248	0.0059	0.6543	0.0059	0.6314
TMAGBM42	0.0892	0.6013	0.0248	0.9958	0.0026	0.6685	0.0011	0.6691	0.0002	0.6698
TMAGBM42	0.0423	0.5987	0.0488	0.9908	0.002	0.6847	0.0009	0.6822	0.0003	0.6757
TMAGBM42	0.0637	0.576	0.0397	0.9932	0.0065	0.6518	0.0075	0.6513	0.0031	0.6427
TMAGBM43	0.0122	0.6004	0.3045	0.9238	0.0005	0.614	0.001	0.6175	0.0126	0.6062
TMAGBM43	0.0081	0.6777	0.0886	0.9902	0.0008	0.7044	0.0002	0.7113	0	0.7016
TMAGBM43	0.0164	0.6326	0.2174	0.9656	0.0011	0.6591	0.0004	0.677	0.0002	0.6666
TMAGBM43	0.0192	0.3626	0.2396	0.6707	0.0028	0.5562	0.0007	0.5745	0.0041	0.5532
TMAGBM43	0.0589	0.5113	0.034	0.9828	0.0008	0.6642	0.0003	0.6701	0.0003	0.6587
TMAGBM44	0.795	0.3583	0.9184	0.4901					0.0147	0.3298
TMAGBM44	0.0651	0.5179	0.3874	0.9421			0.0026	0.7103	0.0021	0.7027

Supplementary Table S3: Results from Inform for each immune marker.

TMA ID	CD68 Positivity	CD68 Tissue Category Area (Tumor %)	CD163 Positivity	CD163 Tissue Category Area (Tumor %)	CD3 Positivity	CD3 Tissue Category Area (Tumor %)	CD4 Positivity	CD5 Tissue Category Area (Tumor %)	CD8 Positivity	CD8 Tissue Category Area (Tumor %)
TMAGBM44	0.0637	0.5532	0.4367	0.9449	0.0079	0.4997	0.0017	0.697	0.0027	0.686
TMAGBM45	0.0771	0.5095	0.7934	0.9596	0.0044	0.6199	0.0007	0.6417	0.0006	0.6457
TMAGBM45	0.0371	0.1208	0.713	0.6089			0.0052	0.64		
TMAGBM45	0.2057	0.1665	0.1034	0.956	0.0221	0.2234	0.012	0.6838	0.0019	0.6673
TMAGBM46	0.0798	0.6429	0.1726	0.9936	0.0027	0.6519	0.002	0.6754	0.0016	0.6718
TMAGBM46	0.071	0.6337	0.0676	0.9913	0.01	0.6229	0.0012	0.675	0.0009	0.6669
TMAGBM46	0.2843	0.4455	0.2279	0.9085	0.0083	0.6506	0.0025	0.6571	0.0017	0.6223
TMAGBM46	0.022	0.3552	0.1828	0.9508	0.0053	0.6124	0.0011	0.6229	0.0014	0.5716
TMAGBM47	0.0276	0.5594	0.0859	0.9793	0.0007	0.6263	0.0002	0.6565	0.0001	0.6504
TMAGBM47	0.0744	0.5234	0.0071	0.9941	0.0012	0.6271	0.0004	0.6297	0.0007	0.6221
TMAGBM47	0.129	0.5545	0.1635	0.993	0.0039	0.6618	0.0007	0.6663	0.0002	0.6688
TMAGBM47	0.2844	0.3116	0.2721	0.9005	0.0225	0.5131	0.0233	0.4785	0.0123	0.4251
TMAGBM47	0.1371	0.6193	0.1075	0.9956	0.0058	0.6722	0.003	0.6862	0.0017	0.6769
TMAGBM48	0.7144	0.213	0.9286	0.8361			0.0102	0.1528	0.0053	0.0839
TMAGBM48	0.0729	0.2148	0.4412	0.3592	0.0209	0.6648	0.0076	0.6817	0.0029	0.668
TMAGBM48	0.1267	0.3064	0.2578	0.4659	0.0031	0.6447	0.0025	0.6719	0.0015	0.6646
TMAGBM49	0.045	0.5598	0.0431	0.9799	0.0016	0.6126	0.0031	0.5988	0.0005	0.591
TMAGBM49	0.049	0.6062	0.0177	0.9919	0.0017	0.6293	0.0016	0.643	0.0003	0.6422
TMAGBM49	0.0207	0.4297	0.1042	0.9297	0.0002	0.5573	0.0022	0.5129	0.0006	0.4208
TMAGBM50	0.1463	0.3265	0.7938	0.7015	0.0052	0.6495	0.0013	0.6619	0.0003	0.6719
TMAGBM50	0.1418	0.3347	0.421	0.7559	0.055	0.59	0.0165	0.6218	0.0049	0.488
TMAGBM50	0.0812	0.236	0.0979	0.9589	0.0026	0.2027	0.0008	0.3786	0.0009	0.3854
TMAGBM51	0.7654	0.5578	1	0.3002	0.0389	0.6584	0.0484	0.6875	0.0104	0.6826
TMAGBM51	0.9413	0.177	0.9403	0.7162	0.0239	0.5852	0.0354	0.6161	0	0.2149
TMAGBM51	0.884	0.2756	0.9259	0.4984	0.0267	0.6	0.0051	0.6308	0.0013	0.3406
TMAGBM52	0.149	0.5915	0.155	0.9665	0.0034	0.6712	0.0018	0.6742	0.0004	0.6623
TMAGBM52	0.0346	0.6148	0.1553	0.9767	0.0051	0.6384	0.0031	0.6649	0.0006	0.6533
TMAGBM52	0.0837	0.4857	0.4362	0.7794	0.0176	0.6749	0.0099	0.6871	0.0002	0.6648
TMAGBM53	0.2972	0.2062	1	0.4058	0.0282	0.5588	0.0248	0.5159	0.0041	0.437
TMAGBM53	0.4353	0.339	1	0.3691	0.0347	0.6074	0.0277	0.6282	0.0028	0.57
TMAGBM53	0.0877	0.3754	0.1967	0.7491	0.0085	0.6226	0.0046	0.6254	0.0014	0.5992
TMAGBM54	0.7016	0.651	1	0.2893	0.0765	0.6621	0.0258	0.6803	0.0287	0.6701
TMAGBM54	0.1703	0.3695	1	0.3387	0.0295	0.6298	0.0136	0.6573	0.0208	0.464
TMAGBM54	0.3803	0.292	0.7073	0.3409	0.0193	0.6785	0.0169	0.6731	0.0089	0.6382
TMAGBM54	0.3401	0.5339	0.8357	0.5285	0.0182	0.6617	0.0069	0.6947	0.0124	0.7031
TMAGBM55	0.1986	0.548	0.7458	0.8636	0.0189	0.5969	0.0172	0.5959	0.004	0.5738
TMAGBM55	0.3601	0.392	0.9212	0.7976	0.0321	0.6242	0.0137	0.6222	0.0072	0.6124

Supplementary Table S3: Results from Inform for each immune marker.

TMA ID	CD68 Positivity	CD68 Tissue Category Area (Tumor %)	CD163 Positivity	CD163 Tissue Category Area (Tumor %)	CD3 Positivity	CD3 Tissue Category Area (Tumor %)	CD4 Positivity	CD5 Tissue Category Area (Tumor %)	CD8 Positivity	CD8 Tissue Category Area (Tumor %)
TMAGBM55	0.5861	0.3382	0.6684	0.5966	0.036	0.3325				
TMAGBM55	0.1166	0.3095	0.6108	0.9148	0.0222	0.6229	0.0119	0.6085	0.0058	0.6243
TMAGBM56	0.147	0.3491	0.446	0.8715	0.0044	0.6249			0.0015	0.6198
TMAGBM56	0.4961	0.2151	0.7323	0.6327	0.0008	0.5883	0.0012	0.5882	0	0.5603
TMAGBM56	0.3282	0.1324	0.3049	0.772	0.0056	0.5025	0.0552	0.2953	0.0019	0.3377
TMA2GBM57	0.6132	0.518	0.9931	0.514	0.0095	0.4814	0.0155	0.4381	0.0128	0.4489
TMA2GBM57	0.179	0.6933	0.9978	0.6749	0.0054	0.6662	0.0241	0.6418	0.0033	0.6628
TMA2GBM57	0.0342	0.5256	0.9682	0.5122	0.009	0.5137	0.0322	0.5228	0.0041	0.5327
TMA2GBM58	0.1116	0.6485	0.6236	0.6299	0.0021	0.639	0.0019	0.6119	0.001	0.6167
TMA2GBM58	0.1639	0.6452	0.9718	0.5949	0.0057	0.6213	0.007	0.5777	0.0023	0.6072
TMA2GBM58	0.2095	0.6697	0.9921	0.6511	0.0045	0.6704	0.0036	0.5943	0.0017	0.6331
TMA2GBM59	0.0223	0.5876	0.24	0.4805	0.0009	0.5371	0.002	0.5513	0	0.5709
TMA2GBM59	0.0354	0.5881	0.0238	0.4887	0.0002	0.5535	0.0048	0.5442	0.0008	0.5629
TMA2GBM59	0.123	0.578	0.0139	0.4889	0.0007	0.556	0.0085	0.556	0	0.5787
TMA2GBM60	0.1591	0.7736	0.7222	0.671	0.0022	0.753	0.0011	0.7371	0.0032	0.7526
TMA2GBM60	0.4869	0.7192	0.9963	0.6247	0.0039	0.6345	0.0134	0.5296	0.0018	0.6417
TMA2GBM60	0.2403	0.6836	0.9991	0.6615	0.0009	0.6618	0.0272	0.6176	0.0003	0.6819
TMA2GBM61	0.1353	0.7064	0.8703	0.6457	0.0055	0.6673	0.0031	0.6556	0.0016	0.6563
TMA2GBM61	0.6393	0.7074	0.9961	0.686	0.0168	0.6843	0.0083	0.6696	0.0091	0.677
TMA2GBM61	0.4818	0.71	0.9848	0.6684	0.0188	0.6924	0.0164	0.6794	0.0082	0.6956
TMA2GBM62	0.1488	0.7264	0.6118	0.0495	0.004	0.6718	0	0.6945	0.0012	0.7053
TMA2GBM62	0.1899	0.7169	0.0807	0.3618	0.0236	0.6682	0.0018	0.6713	0.0099	0.6883
TMA2GBM62	0.056	0.6369	0.8381	0.5689	0.0018	0.5891	0	0.5797	0.0003	0.6058
TMA2GBM63	0.1183	0.6952	0.2268	0.5909	0.0051	0.6589	0.0038	0.6568	0.0042	0.6612
TMA2GBM63	0.0425	0.7267	0.066	0.501	0.0032	0.6795	0.0047	0.673	0.0016	0.6913
TMA2GBM63	0.1346	0.5578	0.4451	0.4824	0.0281	0.3758	0.0521	0.3567	0.0247	0.3146
TMA2GBM64	0.1365	0.6417	0.9992	0.5271	0.0007	0.558	0.0507	0.5292	0.0019	0.5993
TMA2GBM64	0.0825	0.5788	0.9951	0.6156	0.0036	0.6414	0.0075	0.5159	0.0017	0.654
TMA2GBM64	0.0301	0.7232	0.9658	0.6866	0.0014	0.6991	0.0027	0.682	0.0002	0.6975
TMA2GBM65	0.4406	0.5844	0.9932	0.5536	0.0098	0.4614	0.0119	0.5011	0.0075	0.4391
TMA2GBM65	0.0676	0.5661	0.3669	0.5332	0.0017	0.6109	0.0023	0.6124	0.0026	0.6345
TMA2GBM65	0.0654	0.4953	0.6347	0.3781	0.0022	0.5013	0.0023	0.4965	0.0004	0.4874
TMA2GBM66	0.2558	0.4997	0.9786	0.4779	0.029	0.3507	0.0287	0.4058	0.0164	0.3203
TMA2GBM66	0.0158	0.7113	0.8363	0.6879	0.0072	0.6834	0.0043	0.6704	0.0017	0.6945
TMA2GBM66	0.0096	0.6901	0.3542	0.642	0.0009	0.6541	0.001	0.6438	0.0005	0.6644
TMA2GBM67	0.105	0.6386	0.4977	0.5339	0.0014	0.4371	0.007	0.5669	0.0022	0.53
TMA2GBM67	0.0839	0.6446	0.8298	0.4733	0.0025	0.4326	0.0057	0.3565	0.0005	0.4996

Supplementary Table S3: Results from Inform for each immune marker.

TMA ID	CD68 Positivity	CD68 Tissue Category Area (Tumor %)	CD163 Positivity	CD163 Tissue Category Area (Tumor %)	CD3 Positivity	CD3 Tissue Category Area (Tumor %)	CD4 Positivity	CD5 Tissue Category Area (Tumor %)	CD8 Positivity	CD8 Tissue Category Area (Tumor %)
TMA2GBM67	0.0865	0.6799	0.9891	0.6403	0.0116	0.6499	0.0063	0.6135	0.0055	0.5413
TMA2GBM68			0.9566	0.1838			0.023	0.0351	0.0936	0.3101
TMA2GBM68	0.2642	0.6569	0.999	0.6218	0.0255	0.6508	0.0088	0.6115	0.0219	0.627
TMA2GBM68	0.2214	0.7036	0.9966	0.6779	0.0104	0.6979	0.0029	0.6645	0.002	0.6812
TMA2GBM69	0.0309	0.6434	0.3594	0.5799	0.0008	0.5926	0.0002	0.6014	0.0006	0.6128
TMA2GBM69	0.0074	0.6536	0.2343	0.614	0.0006	0.601	0.0006	0.601	0.0002	0.6408
TMA2GBM69										
TMA2GBM70	0.0088	0.6955	0.01	0.4689	0.0007	0.6292	0.0008	0.6517	0.0004	0.6348
TMA2GBM70	0.0679	0.7068	0.0446	0.5234	0.0018	0.6768	0.0017	0.6704	0.0016	0.6778
TMA2GBM70	0.0076	0.6994	0.0108	0.4854	0.0002	0.6659	0.0004	0.6694	0	0.6734
TMA2GBM71	0.0808	0.6203	0.9911	0.5442	0.0519	0.5867	0.0155	0.5362	0.0188	0.513
TMA2GBM71	0.0733	0.6936	0.7314	0.5893	0.0198	0.6827	0.0034	0.6522	0.0202	0.6601
TMA2GBM71	0.138	0.6952	0.988	0.6405	0.0052	0.6599	0.0016	0.6507	0.0014	0.655
TMA2GBM72	0.0413	0.6414	0.2786	0.4863	0.0011	0.634	0.0003	0.6316	0.0016	0.6313
TMA2GBM72	0.1673	0.5898	0.0679	0.1695	0.0013	0.6854	0.0008	0.6923	0.0021	0.7015
TMA2GBM72	0.0242	0.7547	0.1348	0.7053	0.004	0.7002	0.0045	0.6852	0.0008	0.7157
TMA2GBM73	0.0999	0.6911	0.0307	0.2509	0.0007	0.6564	0.0007	0.652	0.0011	0.6613
TMA2GBM73	0.0946	0.5608	0.0229	0.5729	0.0012	0.4542	0.0335	0.3465	0.0008	0.4201
TMA2GBM73	0.3357	0.4118	0.0443	0.25	0.0085	0.4494	0.0031	0.4115	0.0067	0.4419
TMA2GBM74	0.1075	0.5628	0.917	0.5221	0.0078	0.4916	0.0035	0.503	0.0026	0.4599
TMA2GBM74	0.2257	0.5951	0.8099	0.5595	0.0074	0.5749	0.0027	0.5382	0.0024	0.5686
TMA2GBM74	0.1578	0.5973	0.8612	0.3286	0.002	0.5645	0.0004	0.5354	0.0032	0.5882
TMA2GBM75	0.3827	0.2399	0.9918	0.1789	0.012	0.1669	0.0449	0.1745	0.0032	0.1715
TMA2GBM75	0.0998	0.6203	0.6184	0.5748	0.0017	0.567	0.0075	0.5572	0	0.5834
TMA2GBM75	0.2144	0.6263	0.9985	0.6159	0.0046	0.6504	0.0036	0.6114	0.0009	0.6476
TMA2GBM76	0.1057	0.7375	0.1323	0.6672	0.0027	0.6866	0.0034	0.6828	0.0015	0.7083
TMA2GBM76	0.0082	0.7074	0.1791	0.6734	0.0009	0.669	0.0026	0.6639	0.0003	0.6943
TMA2GBM76	0.1896	0.6157	0.695	0.514	0.0002	0.6518	0.0009	0.6285	0	0.6563
TMA2GBM77	0.1034	0.6822	0.0194	0.425	0.0008	0.6539	0.0027	0.6284	0.0007	0.6578
TMA2GBM77	0.1147	0.6637	0.0531	0.2543	0.0028	0.6399	0.0009	0.6265	0.0006	0.6424
TMA2GBM77	0.0746	0.7199	0.0059	0.5872	0.0033	0.6853	0.0018	0.6843	0.0011	0.6883
TMA2GBM78	0.0671	0.5602	0.2063	0.5093	0.0024	0.538	0.0002	0.5643	0.0023	0.5233
TMA2GBM78	0.0461	0.673	0.2027	0.6771	0.001	0.6756	0.0002	0.6549	0.0019	0.6366
TMA2GBM78	0.0608	0.5629	0.2713	0.5464	0.0004	0.5466	0	0.5383	0.0005	0.5116
TMA2GBM79	0.0802	0.6284	0.0785	0.4342	0.0028	0.6182	0.0013	0.5961	0.0022	0.5843
TMA2GBM79	0.0886	0.6296	0.4905	0.6023	0.0031	0.6094	0.001	0.5598	0.0045	0.5798
TMA2GBM79	0.0384	0.6019	0.2771	0.5447	0.0034	0.6168	0.0008	0.5881	0.003	0.5689

Supplementary Table S3: Results from Inform for each immune marker.

TMA ID	CD68 Positivity	CD68 Tissue Category Area (Tumor %)	CD163 Positivity	CD163 Tissue Category Area (Tumor %)	CD3 Positivity	CD3 Tissue Category Area (Tumor %)	CD4 Positivity	CD5 Tissue Category Area (Tumor %)	CD8 Positivity	CD8 Tissue Category Area (Tumor %)
TMA2GBM80	0.2131	0.6368	0.0651	0.4895	0.0004	0.6552	0.0017	0.6529	0.0014	0.6031
TMA2GBM80	0.0098	0.4666	0.1188	0.3139	0.0004	0.4109	0.0012	0.4329	0	0.3692
TMA2GBM80	0.006	0.6362	0.0966	0.1038	0.0002	0.6515	0	0.6487	0	0.5987
TMA2GBM81	0.0482	0.3285	0.2639	0.6349	0	0.5666	0	0.6537	0.0018	0.4302
TMA2GBM81	0.0748	0.6201	0.2106	0.6057	0.0011	0.5719	0.0023	0.5637	0.004	0.6018
TMA2GBM81	0.0548	0.6461	0.3735	0.648	0.0015	0.6724	0.001	0.6738	0.0026	0.6249
TMA2GBM82	0.0513	0.6712	0.9141	0.6701	0.0012	0.6787	0.0002	0.6654	0.0004	0.6495
TMA2GBM82	0.0341	0.4871	0.9989	0.5911	0.0016	0.5388	0	0.5783	0.0008	0.5212
TMA2GBM82	0.0242	0.5907	0.9972	0.6464	0.0035	0.622	0.0024	0.6402	0.0031	0.6065
TMA2GBM83	0.0334	0.721	0.0952	0.7349	0.0033	0.7319	0.0006	0.7089	0.0055	0.6932
TMA2GBM83	0.1115	0.6154	0.0881	0.5063	0.0033	0.6368	0.0009	0.6285	0.007	0.5951
TMA2GBM83	0.1358	0.6333	0.0321	0.4032	0.0028	0.642	0.0004	0.6298	0.0015	0.6016
TMA2GBM84	0.0578	0.592	0.2359	0.595	0.0012	0.622	0.001	0.6121	0.0024	0.595
TMA2GBM84	0.0761	0.6098	0.8237	0.6526	0.0018	0.6579	0.0012	0.6493	0.0011	0.6237
TMA2GBM84	0.0569	0.6444	0.4412	0.6664	0.0002	0.6525	0.0002	0.6439	0.0011	0.623
TMA2GBM85	0.0258	0.5681	0.0963	0.5668	0.0062	0.6343	0.0004	0.6211	0.0046	0.5951
TMA2GBM85	0.0358	0.6032	0.1836	0.1986	0.003	0.626	0.0009	0.5919		
TMA2GBM85	0.0203	0.4614	0.0641	0.4052	0.0062	0.4776	0.0019	0.4632	0.0068	0.4606
TMA2GBM86	0.0286	0.5552	0.1498	0.5351	0.0009	0.5826	0.0027	0.5736	0.0003	0.5539
TMA2GBM86	0.1924	0.4737	0.1546	0.3998	0.0179	0.4926	0.0083	0.4976	0.0098	0.4783
TMA2GBM86	0.0421	0.5183	0.8077	0.5935	0.0088	0.5986	0.0381	0.5795	0.0015	0.5688
TMA2GBM87	0.1014	0.6871	0.5949	0.6711	0.0004	0.6611	0.0003	0.6581	0.0142	0.635
TMA2GBM87	0.1057	0.5909	0.9967	0.589	0.0196	0.5847	0.0038	0.5902	0.0203	0.5682
TMA2GBM87	0.0434	0.2419	0.8385	0.2481	0.0188	0.2597	0.0133	0.2393	0.0158	0.2511
TMA2GBM88	0.1177	0.6601	0.3871	0.6926	0.0048	0.6924	0.0017	0.7169	0.0006	0.6577
TMA2GBM88	0.5302	0.4397	0.9838	0.4825	0.0027	0.5511	0.0039	0.5637	0.0124	0.4701
TMA2GBM88	0.1338	0.4659	0.624	0.5747	0.0031	0.5715	0.0056	0.5639	0.0103	0.5458
TMA2GBM89	0.1294	0.6271	0.6974	0.6187	0.003	0.6303	0.0009	0.6297	0.0045	0.5895
TMA2GBM89	0.12	0.592	0.4541	0.618	0.0055	0.6314	0.0014	0.6316	0.0058	0.5859
TMA2GBM89	0.1637	0.5194	0.9037	0.5893	0.0109	0.5917	0.0019	0.5872	0.0076	0.5656
TMA2GBM90	0.4764	0.3471	0.9244	0.6026	0.0096	0.5932	0.0062	0.5632	0.0079	0.5588
TMA2GBM90	0.5468	0.4319	0.8238	0.624	0.0244	0.561	0.0295	0.5998	0.0312	0.5701
TMA2GBM90	0.298	0.4889	0.9103	0.6521	0.0204	0.6435	0.0028	0.6403	0.0206	0.6228
TMA2GBM91	0.163	0.4227	0.9399	0.5387	0.0042	0.4975	0.0015	0.4959	0.0057	0.4225
TMA2GBM91	0.1716	0.4541	0.3521	0.6107	0.0165	0.6239	0.002	0.6412	0.0089	0.5952
TMA2GBM91	0.6905	0.5334	0.9991	0.6314	0.0031	0.646	0.0011	0.6366	0.0043	0.6222
TMA2GBM92	0.0665	0.5405	0.9922	0.5616	0.0074	0.6287	0.0023	0.6167	0.0043	0.5841

Supplementary Table S3: Results from Inform for each immune marker.

TMA ID	CD68 Positivity	CD68 Tissue Category Area (Tumor %)	CD163 Positivity	CD163 Tissue Category Area (Tumor %)	CD3 Positivity	CD3 Tissue Category Area (Tumor %)	CD4 Positivity	CD5 Tissue Category Area (Tumor %)	CD8 Positivity	CD8 Tissue Category Area (Tumor %)
TMA2GBM92	0.0357	0.621	0.3906	0.6336	0.0005	0.6382	0.0007	0.6432	0.0013	0.5995
TMA2GBM92	0.1674	0.6681	0.9384	0.6632	0.0029	0.682	0.0011	0.678	0.0055	0.6331
TMA2GBM93	0.1177	0.6797	0.028	0.0939	0	0.6898	0.0005	0.7	0.0023	0.6778
TMA2GBM93	0.0236	0.6387	0.0161	0.4431	0.0026	0.6712	0.0008	0.6776	0.0016	0.6518
TMA2GBM93	0.1936	0.5688	0.0332	0.2329	0.0003	0.634	0.0006	0.6479	0.0022	0.6193
TMA2GBM94	0.8081	0.483	0.944	0.1182	0.0166	0.0525			0	0.0249
TMA2GBM94	0.4736	0.1526	0.9452	0.3641	0.0016	0.2862	0.0088	0.2658	0.0046	0.3266
TMA2GBM94	0.1293	0.3788	0.9691	0.4347	0.0038	0.2907	0.0099	0.3636	0.0085	0.2475
TMA2GBM95	0.3022	0.5137	0.1578	0.49	0.004	0.6378	0.0007	0.561	0.0043	0.6228
TMA2GBM95	0.5003	0.6086	0.6306	0.6261	0.011	0.6666	0.0014	0.6564	0.0069	0.647
TMA2GBM95	0.1942	0.6246	0.6336	0.6593	0.005	0.6577	0.0065	0.6519	0.0298	0.6265
TMA2GBM96	0.1002	0.3225	0.5603	0.3118	0.0018	0.5842	0.0015	0.5351	0.0011	0.5653
TMA2GBM96	0.2148	0.5183	0.9199	0.5278	0.003	0.6034	0	0.6144	0.0021	0.5843
TMA2GBM96	0.0414	0.6168	0.0941	0.2354	0.0031	0.6429	0.0007	0.6401	0.0014	0.6111
TMA2GBM97	0.0628	0.57	0.8021	0.5888	0.0019	0.5844	0.0043	0.6015	0.002	0.5795
TMA2GBM97	0.1018	0.6372	0.3617	0.6365	0.0096	0.6658	0.0025	0.6593	0.0103	0.6379
TMA2GBM97	0.1244	0.5106	0.9045	0.6641	0.0006	0.6433	0.0101	0.6491	0.0045	0.6214
TMA2GBM98	0.2053	0.6725	0.9977	0.7028	0.0144	0.7032	0.0027	0.708	0.0417	0.6658
TMA2GBM98	0.0619	0.6757	0.5543	0.678	0.0041	0.7017	0.002	0.6951	0.0016	0.6507
TMA2GBM98	0.1631	0.6156	0.9178	0.6584	0.0069	0.6517	0.0031	0.6451	0.0027	0.6155
